# Simulation models predict that school-age children are responsible for most human-to-mosquito *Plasmodium falciparum* transmission in southern Malawi

**DOI:** 10.1186/s12936-018-2295-4

**Published:** 2018-04-03

**Authors:** Jenna E. Coalson, Lauren M. Cohee, Andrea G. Buchwald, Andrew Nyambalo, John Kubale, Karl B. Seydel, Don Mathanga, Terrie E. Taylor, Miriam K. Laufer, Mark L. Wilson

**Affiliations:** 1 Department of Epidemiology, School of Public Health, University of Michigan, M5507 SPH II, 1415 Washington Heights, Ann Arbor, MI 48109 USA; 2 Division of Malaria Research, Institute of Global Health, University of Maryland School of Medicine, Baltimore, MD USA; 3 Blantyre Malaria Project, University of Malawi College of Medicine, Blantyre, Malawi; 4 College of Osteopathic Medicine, Michigan State University, East Lansing, USA; 5 Malaria Alert Centre, University of Malawi College of Medicine, Blantyre, Malawi; 6 Present Address: Center for Insect Science and Department of Epidemiology and Biostatistics, University of Arizona, Tucson, AZ USA

**Keywords:** Malaria, Transmission reservoirs, School-age children, Simulation model, *Plasmodium falciparum*, Gametocytes

## Abstract

**Background:**

Malaria persists in some high-transmission areas despite extensive control efforts. Progress toward elimination may require effective targeting of specific human populations that act as key transmission reservoirs.

**Methods:**

Parameterized using molecular-based *Plasmodium falciparum* infection data from cross-sectional community studies in southern Malawi, a simulation model was developed to predict the proportions of human-to-mosquito transmission arising from (a) children under 5 years old (U5s), (b) school-age children (SAC, 5–15 years), (c) young adults (16–30 years), and (d) adults > 30 years. The model incorporates mosquito biting heterogeneity and differential infectivity (i.e. probability that a blood-fed mosquito develops oocysts) by age and gametocyte density.

**Results:**

The model predicted that SAC were responsible for more than 60% of new mosquito infections in both dry and rainy seasons, even though they comprise only 30% of this southern Malawi population. Young adults were the second largest contributors, while U5s and adults over 30 were each responsible for < 10% of transmission. While the specific predicted values are sensitive to the relative infectiousness of SAC, this group remained the most important contributor to mosquito infections under all realistic estimates.

**Conclusions:**

These results suggest that U5 children play a small role compared to SAC in maintaining *P. falciparum* transmission in southern Malawi. Models that assume biting homogeneity overestimate the importance of U5s. To reduce transmission, interventions will need to reach more SAC and young adults. This publicly available model can be used by others to estimate age-specific transmission contributions in epidemiologically similar sites with local parameter estimates of *P. falciparum* prevalence and bed net use.

**Electronic supplementary material:**

The online version of this article (10.1186/s12936-018-2295-4) contains supplementary material, which is available to authorized users.

## Background

Although malaria control interventions have reduced parasite prevalence in some regions, ambitious efforts have caused little reduction in many settings with high endemicity [1–6]. Progress toward elimination requires understanding why current strategies fail. One potential explanation is that they do not adequately target sources of human-to-mosquito *Plasmodium* transmission. Defining the primary human reservoirs of transmission, or people who contribute most to infecting competent vectors, requires an understanding of infection patterns in human populations, variations in infectivity when bitten, and differential likelihoods of being bitten by female *Anopheles*.

Southern Malawi is one area where, despite high coverage of insecticide-treated nets (ITNs), the malaria burden remains high. Recent cross-sectional studies have found that 5–15-year old, school-age children (SAC) have the highest prevalence of *Plasmodium falciparum* infection [7], are more likely to carry gametocytes when parasitaemic [8], and are less likely to use bed nets [9] than children under five (U5s) and adults over 15 years. These findings suggest that SAC may be important human-to-mosquito transmission reservoirs in that area, but their contribution has not been quantified.

Several skin- or membrane-feeding studies in other sites have calculated age-specific contributions of human infectious reservoir populations [10–18]. While they elucidated the difference between *infected* and *infectious* humans, most models did not consider whether mosquito feeding heterogeneity influences which people are key *transmission* reservoirs. Indeed, highly infectious people who are never bitten cannot contribute to transmission, while weakly infectious people who are frequently bitten might contribute considerably. Mosquito feeding frequency has been found to increase with the age of human hosts, possibly due in part to increasing body surface area [18–22]. Recently, Gonçalves et al. [18] matched mosquito blood meals to their human sources and found evidence of extreme heterogeneity in biting frequency by age. In the high-transmission setting of Burkina Faso, adults were bitten 20 times as often as U5s, and SAC were bitten 7 times as often as U5s, and, accordingly, the adult contribution to transmission was underestimated when considering only the proportion of infectious people by age [18]. Assuming blood-feeding homogeneity limits the estimation of true transmission contributions.

The present study aimed to estimate human, age-specific transmission of *P. falciparum* to mosquitoes, based on 3 years of surveillance data from southern Malawi, using a model that incorporated heterogeneity in mosquito exposure to different age-groups due to differential ITN use and likelihood of being bitten. The hypothesis was that SAC are the primary contributors to transmission, particularly during the dry season. A secondary aim was to develop a simple modelling tool to enable researchers or policy-makers in other settings to evaluate relative transmission by different human groups using key surveillance data on parasite prevalence and ITN use.

## Methods

### Ethics, consent, and permissions

The human infection data used in this model were derived from repeated cross-sectional studies in southern Malawi. Informed consent was obtained from all participants or their guardians; assent was also obtained from 13 to 17 year old participants. The study protocol was approved by the independent Institutional Review Boards of the University of Malawi College of Medicine, the University of Maryland, Baltimore, and Michigan State University.

### Study setting

Southern Malawi has perennially endemic malaria, with elevated incidence during the rainy season [3]. *P. falciparum* is predominant, though *Plasmodium ovale* and *Plasmodium malariae* infections also occur (typically as co-infections with *P. falciparum*) [23]. Cross-sectional data were gathered at the ends of the dry and rainy seasons from 2012 to 2015 from sites sampled in three ecologically distinct districts: urban highlands with relatively low transmission, rural highlands with moderate transmission, and rural lowlands with high transmission.

### Model and analysis

**Fig. 1:**
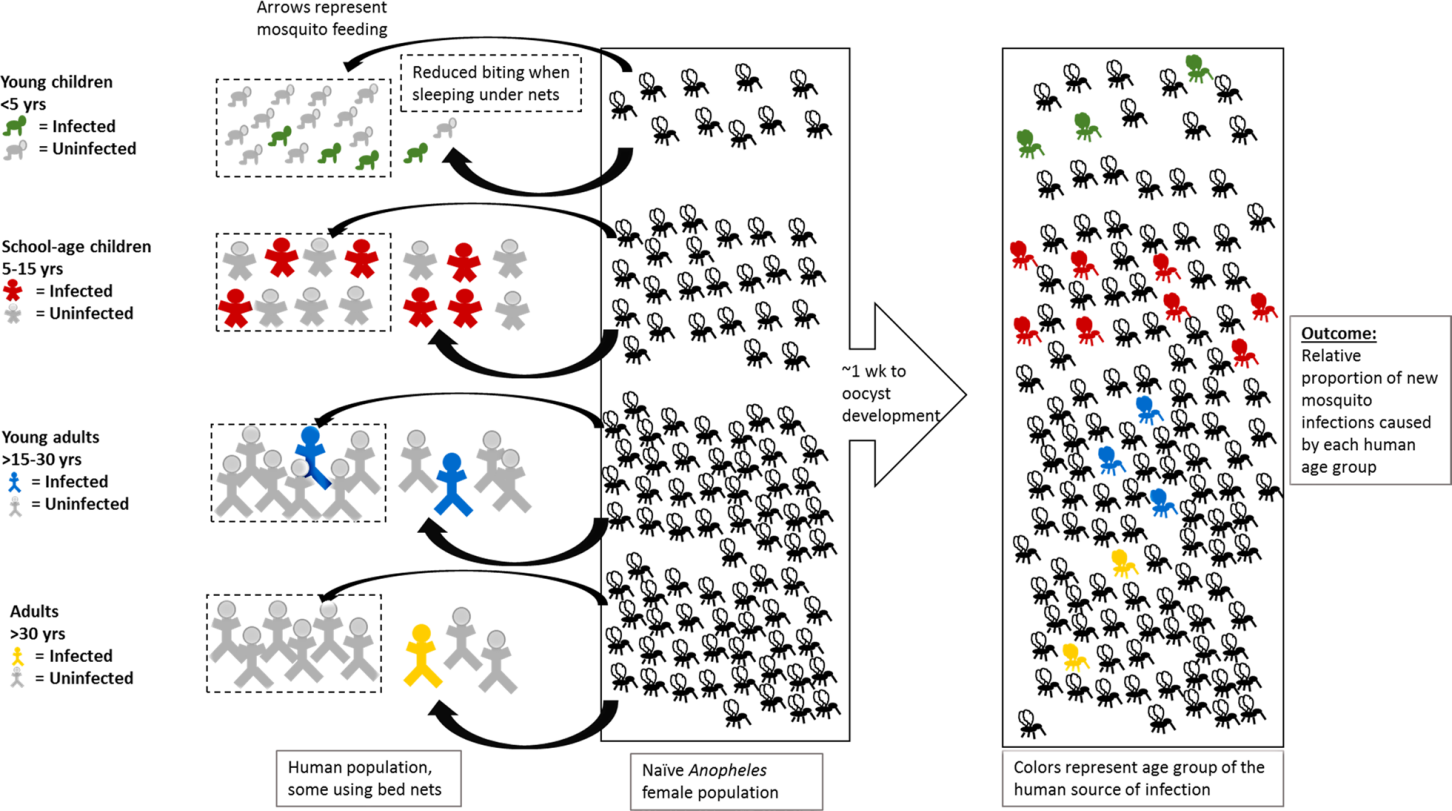
Graphical depiction of model. *Anopheles* mosquitoes feed on humans with a relative frequency that depends on age and bed net use (dotted lines). The likelihood that a person carries *P. falciparum* parasites (colored figures) depends on age, and the likelihood that a blood meal leads to an oocyst infection in the mosquito (colored mosquitoes) is influenced by the distribution of gametocyte densities in infections in each age group. The model ultimately estimates the proportion of new mosquito infections that can be attributed to having fed on humans in each age group

A mathematical model was developed to estimate the proportion of new mosquito infections attributable to people in four age categories: (1) young children, < 5 years; (2) SAC, 5–15 years; (3) young adults, > 15–30 years; and (4) adults, > 30 years (Fig. 1). Their contributions were estimated statically for the ends of the dry and rainy seasons.

Estimating the number of new mosquito infections attributable to each age group, *M*_*a*_, involved three components: (1) the number of people in each age group with *P. falciparum* infection, *n*_*a*_, (2) the probability of human-to-mosquito transmission in a single blood meal from an infected person, or human infectivity, *κ*, and (3) the relative number of blood meals taken per human age group, *b*_*a*_. Human-to-mosquito infectivity, κ, was considered dependent on gametocyte density (*d*) in all iterations of the model output, with additional effects of age (*a*) in some sensitivity analyses.

As a simplifying assumption, the population was divided into four categories of age and four categories of gametocyte density, for a total of 16 compartments. Densities were categorized as: (1) less than one gametocyte per μL, (2) one to 200 gametocytes per μL, (3) 201–400 gametocytes per μL, and (4) more than 400 gametocytes per μL [24]. The number of people within each of the 16 age and gametocyte density categories, *n*_*a*,*d*_, was calculated from the total number of people in the age group, *N*_*a*_, based on census data, the prevalence of *P. falciparum* infection, *α*_*a*_, in age group *a* estimated by quantitative polymerase chain reaction (qPCR), and the proportion of qPCR-positive infections in the age group in which the density of gametocytes is *d*, *g*_*a*,*d*_, as:

1 $$n_{a ,d} = N_{a} \alpha_{a} g_{a ,d}$$

Differential prevalence of infection by age may be due to risk of becoming infected or of infection duration, the latter being influenced by acquired immune responses and variation in treatment-seeking. Since our model was static, and calculated reservoir contributions for certain times of the year based on measured prevalences, these prevalence parameters were used without disentangling the root causes.

Human-to-mosquito infectivity was assumed in all iterations of the model to increase with gametocyte density, *κ*_*d*_ [24–26]. Differences in infectivity by age, *κ*_*a*_, that are independent of the effect of gametocyte density are difficult to measure and poorly defined, but may be influenced by recent treatments, transmission-blocking immunity, and other unknown factors. Accordingly, estimates from the literature were used to establish the best estimates for baseline values and to estimate reasonable ranges for a sensitivity analysis.

The relative biting frequency by age, *b*_*a*_, was calculated from: (1) estimated relative biting preference by age, *δ*_*a*_, as used by Stone et al. [21], (2) the prevalence of ITN use in each age group, *σ*_*a*_, and (3) the relative reduction in biting frequency attributable to ITN use, *γ*, as:

2 $$b_{a} = \delta_{a} \left( {\sigma_{a} \gamma + \left( {1 - \sigma_{a} } \right)} \right)$$

The number of new mosquito infections attributable to each age group, *M*_*a*_ was then calculated as:

3 $$M_{a} = \kappa_{a} b_{a} \sum\limits_{d = 1}^{4} {\left( {n_{a ,d} k_{d} } \right)}$$

As several parameters were calculated as relative estimates across age groups rather than as exact quantifications, the results for *M*_*a*_ were used to calculate the proportion of new mosquito infections attributable to each age group, *F*_*a*_:

4 $$F_{a} = \frac{{M_{a} }}{{\sum_{a = 1}^{4} {M_{a} } }}$$

Brief descriptions and sources of data for each parameter are provided in Table 1.

**Table 1:** Model parameter description and data sources

Parameter	Description	Source	Value
*n*_*a*,*d*_	Number of humans of age group *a* that are *P. falciparum*-infected with gametocytes at density *d* in Southern Malawi	Calculated, Eq. 1	$n_{a ,d} = N_{a} \alpha_{a} g_{a ,d}$
*N*_*a*_	Number of people in age group *a* in Southern Malawi	2008 Census [27]	
*α*_*a*_	Prevalence of *P. falciparum* in age group *a*	ICEMR-Malawi primary data	See Table 2
*g*_*a*,*d*_	Proportion of infections in age group *a* with gametocyte density *d*	ICEMR-Malawi primary data	See Table 2
*κ*_*d*_	Percent of mosquitoes infected with oocysts after a blood meal from a person with gametocytes at density *d*	Churcher et al. [24]	0.015 *d* = 1 (< 1/μL) 0.04 *d* = 2 (1–200/μL) 0.10 *d* = 3 (> 200–400/μL) 0.18 *d* = 4 (> 400/μL)
*κ*_*a*_	Relative infectivity per blood meal by age category *a*	Ouédraogo et al. [17] (including personal data communication)	2.5 *a* = 1 (< 5 years) 1.7 *a* = 2 (5–15 years) 1.0 (ref) *a* = 3 (> 15–30 years) 0.6 *a* = 4 (> 30 years)
*b*_*a*_	Relative mosquito biting frequency by human age group *a*	Calculated, Eq. 2	$b_{a} = \delta_{a} \left( {\sigma_{a} \gamma + \left( {1 - \sigma_{a} } \right)} \right)$
*δ*_*a*_	Vector’s relative feeding preference for human age group *a*	Stone et al. [21]	1.0 (ref) *a* = 1 (< 5 years) 3.0 *a* = 2 (5–15 years) 4.0 *a* = 3 (> 15–30 years) 4.0 *a* = 4 (> 30 years)
*g*_*a*_	Proportion of people in age group *a* who use a bed net	ICEMR-Malawi primary data	See Table 2
*γ*	Relative reduction in nightly biting frequency for bed net users compared to non-users	Killeen et al. [28]	0.40
*M*_*a*_	Relative number of new mosquito oocyst infections attributable to human age group *a*	Calculated, Eq. 3	$M_{a} = \kappa_{a} b_{a} \sum\limits_{d = 1}^{4} {\left( {n_{a ,d} k_{d} } \right)}$
*F*_*a*_	Fraction of all new mosquito infections attributable to human age group *a*	Calculated, Eq. 4	$F_{a} = \frac{{M_{a} }}{{\sum_{a = 1}^{4} {M_{a} } }}$

*ICEMR* International Center of Excellence for Malaria Research

### Study design for primary data collection

Reported ITN use, *σ*_*a*_, and molecular data characterizing parasite prevalence, *α*_*a*_, by age for both seasons were obtained from cross-sectional studies carried out every 6 months from 2012 to 2014; data on gametocyte density distribution by age and season, *g*_*a*,*d*_, were obtained from additional surveys that occurred in a subset of sites in the rainy and dry seasons of 2015.

#### Parasite prevalence and net use

The design of the original six surveys has been previously reported [7, 9]; briefly, clusters of 30 households within ten enumeration areas were chosen by cluster-random sampling in each of the three study districts. All present and consenting members of these ~ 900 households were surveyed regarding malaria symptoms, treatments, and ownership/use of ITNs. Participants over 6 months of age had finger-prick blood sampled for qPCR. qPCR was run in singlicate with a limit of detection of 2.7 parasites per μL; *P. falciparum* infection was considered to be present if the sample produced a positive curve for Pf lactate dehydrogenase (LDH) [7, 29]. Molecular data on gametocyte density were not available for the samples taken from 2012 to 2014, so the distribution of gametocyte densities into the four density compartments by age category and season were predicted using molecular gametocyte data collected in 2015, as described in the next section.

#### Gametocyte density distribution

In 2015, four of the sites from the two rural districts were chosen for an extension study to schools. For each site, members of the original 30 households were included with an additional 50 households and cohorts of ~ 100 students in the nearest primary school. All participants provided finger-prick blood on filter paper for qPCR and preserved in RNAprotect^®^ (Qiagen Inc., Valencia, CA) for quantitative reverse transcription PCR (qRT-PCR).

Gametocyte density distributions were measured among *P. falciparum* carriers from the 2015 household samples and the baseline school cohort samples. qPCR targeting LDH was run in duplicate to screen for infection. If either well was positive, qRT-PCR was performed to detect the mature gametocyte marker *Pfs25* [25, 30–32]. Gametocyte density was quantified using a standard curve derived from cultured and diluted Pf.2004 TdT *P. falciparum*. Additional detail on gametocyte testing methods is provided as Additional file 1.

For all PCR-positive infections, the age- and season-specific distributions of gametocyte densities were determined for the four gametocyte density categories defined above; some age- and season-specific compartments were empty. One individual was added to all compartments to smooth distribution estimates for the model.

The distribution of gametocyte density category by age and season in 2015 was assumed to apply to qPCR-positive infections from the 2012 to 2014 surveys, for which gametocyte data were not available. Total parasite density was significantly correlated with gametocyte density in 2015 (p = 0.03), and no significant difference was found in the association of age and total parasite density in the 2012–2014 dataset versus the 2015 dataset for either the rainy or dry season, supporting the assumption that the distribution of gametocyte densities by age and season were similar among PCR-positive cases in the two datasets.

### Secondary data for estimation of other parameter values

The population distribution for Malawi’s Southern Region was abstracted from the most recent census in 2008 [27]. Published journal articles guided estimation of human-to-mosquito infectivity based on age and gametocyte density, differential mosquito biting by age, and reduction in biting frequency associated with ITNs (Table 1).

Comparisons were made for predicted transmission contributions when assuming homogeneous biting, as in previous reports, to those that allowed for differential biting by age and ITN use. This model used the same estimates of relative biting as Stone et al. [21] based on differences in body surface area. The values are consistent with data from human landing catch studies, and may even underestimate the increase with age [18–22].

Investigators have researched human-to-mosquito infectivity for decades, but the determining factors remain controversial and poorly quantified (a summary of age-related infectivity data is provided as Additional file 2). It is widely accepted that infectivity increases with gametocyte density, though the exact shape of the association is still debated [24–26], so all model runs adjusted for a density-dependent infectivity parameter. Published studies generally indicate that infectiousness decreases with age, though methods vary widely, sample sizes tend to be small, few adjusted for gametocyte density, and findings are inconsistent [10–17, 33–43]. Data stratified by age and gametocyte status (i.e. microscopic versus submicroscopic) from a recent transmission study in Burkina Faso were used to estimate the baseline parameters for infectivity by age beyond the effects of gametocyte density in the present model, (*κ*_*a*_), as 2.5:1.7:1.0:0.6 for U5s, SAC, young adults, and adults > 30, respectively (Table 1) [17]. Sensitivity analyses were carried out within a reasonable range of estimates of infectivity. A few studies reported slightly lower infectiousness for U5s and SAC than for adults [11, 33, 34], so the lower bound of *κ*_*a*_ was set to 0.75 for both groups (relative to young adults). The upper bound for both was set to 4.0, as no published data supported a higher difference in infectivity by age after accounting for gametocyte density.

## Results

### Input data

SAC had the highest parasite prevalence in both seasons (Table 2). Using age distributions from the 2008 census, an estimated 12% of *P. falciparum* carriers at the end of the dry season were U5s, 50% were SAC (5–15 years), 26% were young adults (16–30 years), and 11% were adults over 30. The pattern was similar, but slightly more evenly distributed, at the end of the rainy season, when SAC comprised 44% of *P. falciparum* infections.

**Table 2:** Population characteristics by season and estimated distribution of human reservoirs of infection

Age group	% of population (*N*_*a*_/*N*) [27] (%)	Dry season			Rainy season		
		*P. falciparum* prevalence (qPCR), *α*_*a*_ (%)	ITN usage, *σ*_*a*_ (%)	% of *P. falciparum* infections^a^ (%)	*P. falciparum* prevalence (qPCR), *α*_*a*_ (%)	ITN usage, *σ*_*a*_ (%)	% of *P. falciparum* infections^a^ (%)
Children (< 5 years)	18.2	7.7	53.4	12.4	13.6	70.1	13.8
SAC (5–15 years)	30.0	18.9	33.6	50.2	26.6	49.0	44.3
Young adults (16–30 years)	27.2	10.8	59.7	26.0	16.3	65.3	24.6
Adults (> 30 years)	24.7	5.2	56.4	11.3	12.7	63.0	17.4

^a^Estimated percent of parasite carriers that are from each age group, (*N*_*a*_/*N*)*α_*a*_

**Fig. 2:**
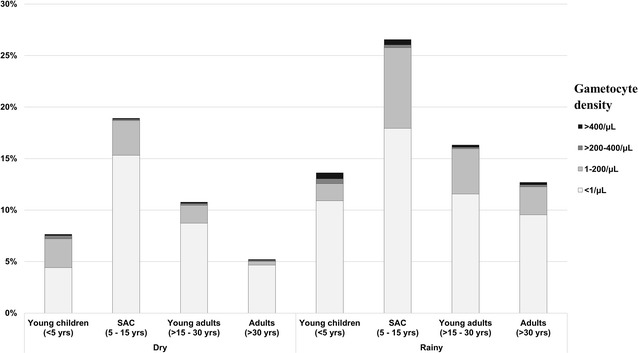
qPCR-based prevalence of *P. falciparum* infection, stratified by qRT-PCR-predicted gametocyte density

Most infections had 0 or < 1 gametocyte/μL detected. Higher densities (> 200 gametocytes/μL) were more common during the rainy season and in children than adults, but comprised a small proportion of infections in both seasons. The predicted distributions are displayed with the average qPCR-based prevalences by age and season in Fig. 2.

### Estimates of transmission contribution and sensitivity to biting homogeneity assumptions

**Fig. 3:**
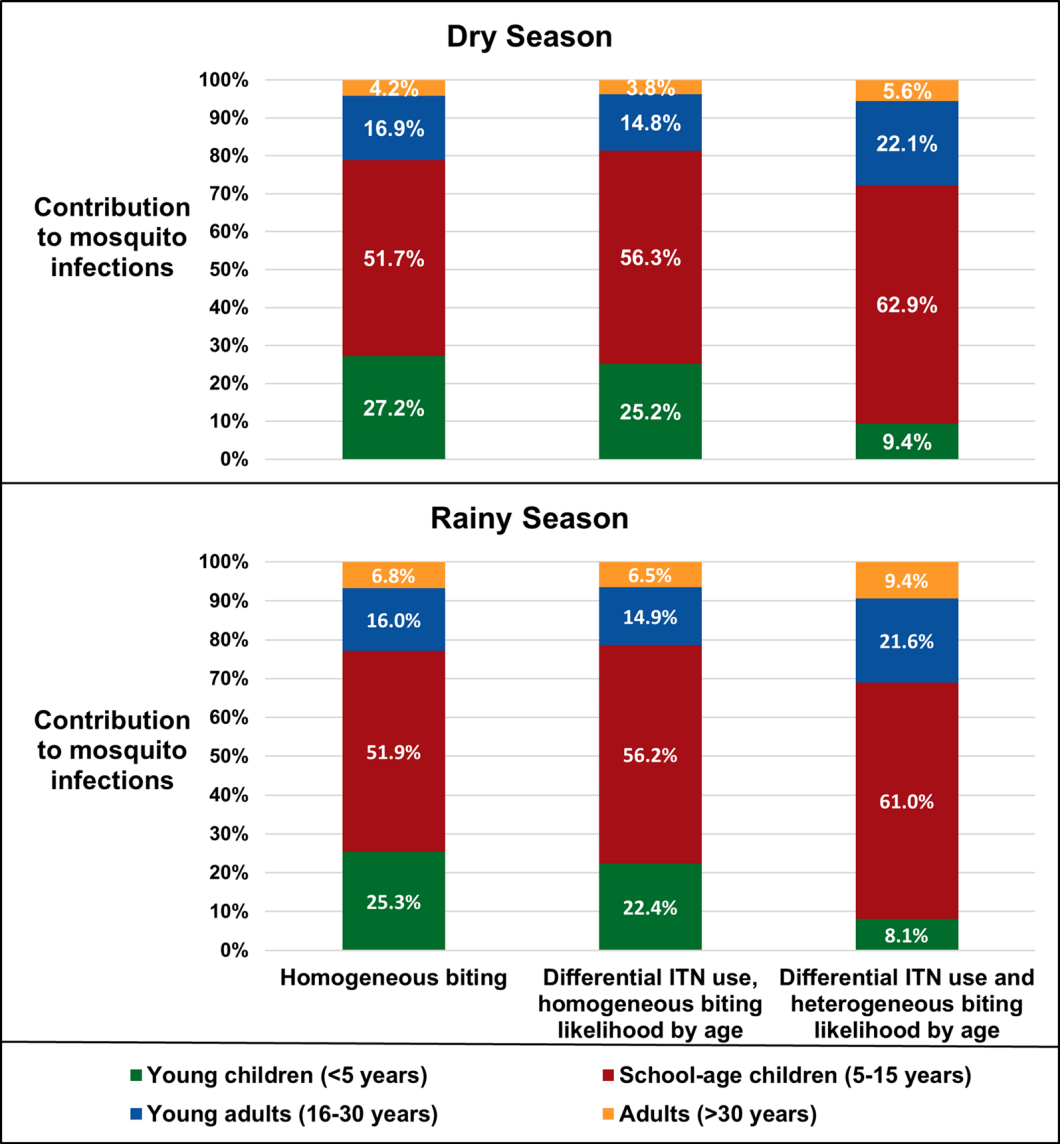
Predicted transmission contributions under different assumptions about mosquito feeding frequency by human age

Figure 3 displays the predicted contributions to transmission by age under three assumptions: (1) mosquito feeding was completely homogeneous by age, as in many prior infectivity studies, (2) ITNs reduced feeding on people using them, but mosquitoes were otherwise equally likely to bite people regardless of age, and (3) ITNs reduced feeding and the biting likelihood differed by age. Notably, there was very little seasonal difference in the age-specific contributions to new mosquito infections under any condition.

Despite comprising only 30% of the population, all three models predicted that SAC caused the majority (> 50%) of human-to-mosquito transmission of *P. falciparum* in southern Malawi in both seasons (Fig. 3). Relaxing the biting homogeneity assumption to account for heterogeneous ITN use and likelihood of biting based on body size increased SAC’s predicted transmission contribution to more than 60%. Adults over 30, who comprised ~ 25% of the population, were estimated to contribute little (< 10%) to new mosquito infections in either season under all heterogeneity assumptions.

Incorporating differential ITN use had less impact than assuming homogeneous likelihood of biting based on age/body size. Assuming homogeneous biting overestimated the contribution of U5s, who have small body surface area and high ITN use, and underestimated the contributions of SAC and young adults. Models that assumed homogeneous biting by age roughly tripled the predicted transmission contribution of U5s (who comprised 18.2% of the population), from 9.4 to 25.2% during the dry season, and 8.1 to 22.4% during the rainy season. Incorporating biting heterogeneity reduced their predicted contributions to < 10% and made young adults, who comprised 27.2% of the population, the second largest transmission contributors at 21.6 and 22.1%.

### Sensitivity analyses for human infectivity to mosquitoes by age

**Fig. 4:**
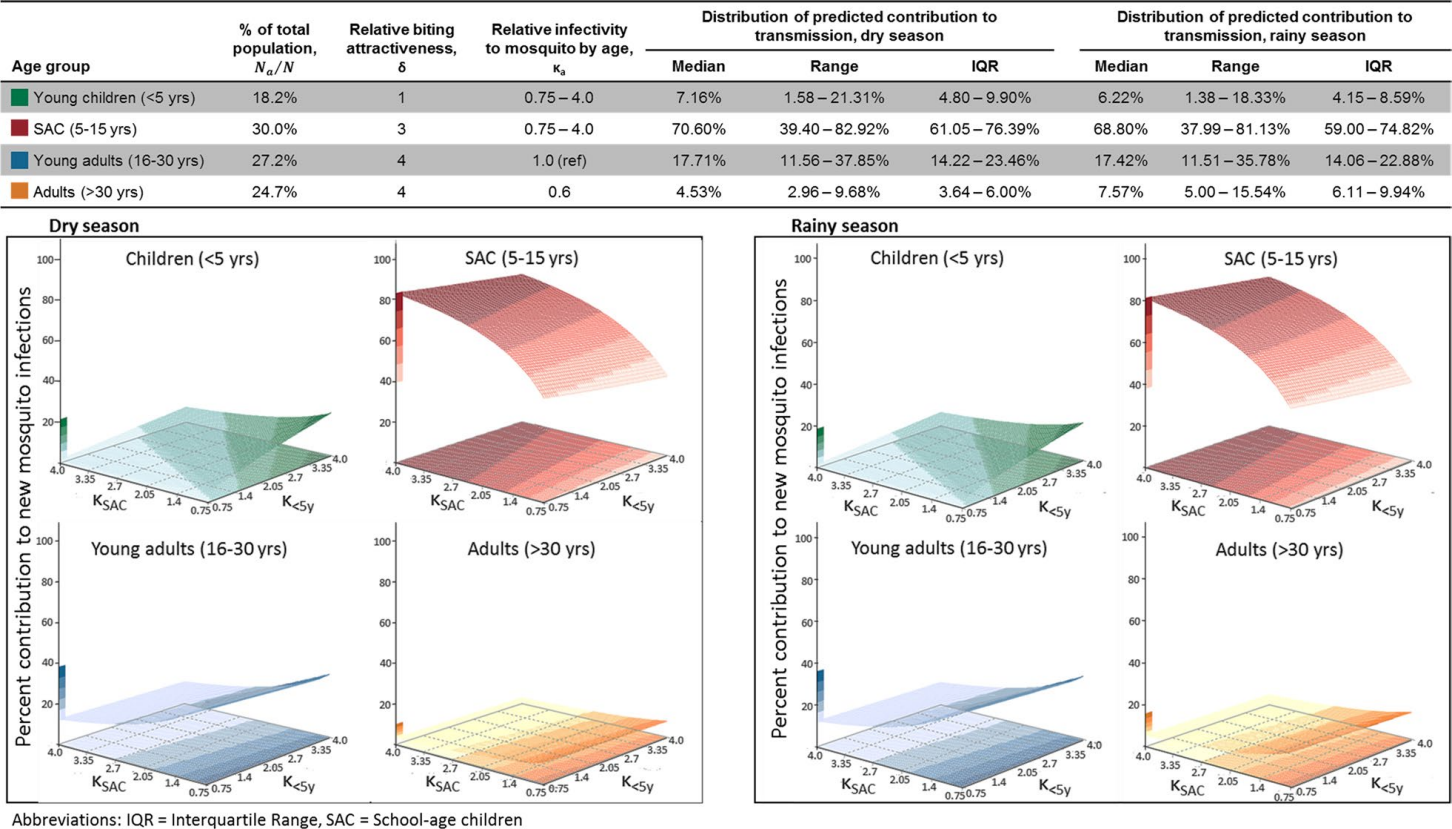
Predicted transmission contributions in a sensitivity analysis varying human-to-mosquito infectivity (*κ*_*a*_) by age. Infectivity to mosquitoes may vary with human age in relation to differences in recent antimalarial treatment, treatment-blocking immunity, duration of infection, multiplicity of infection, or other unknown factors, all of which could differ with age, even after accounting for gametocyte presence and density. Thus a sensitivity analysis was performed for transmission contribution when varying infectivity by age with outside bounds informed by the scientific literature on membrane feeding experiments that accounted for gametocyte status. Infectivity of children under 5 years and school-age children were varied relative to the infectivity of young adults (16–30 years)

**Fig. 5:**
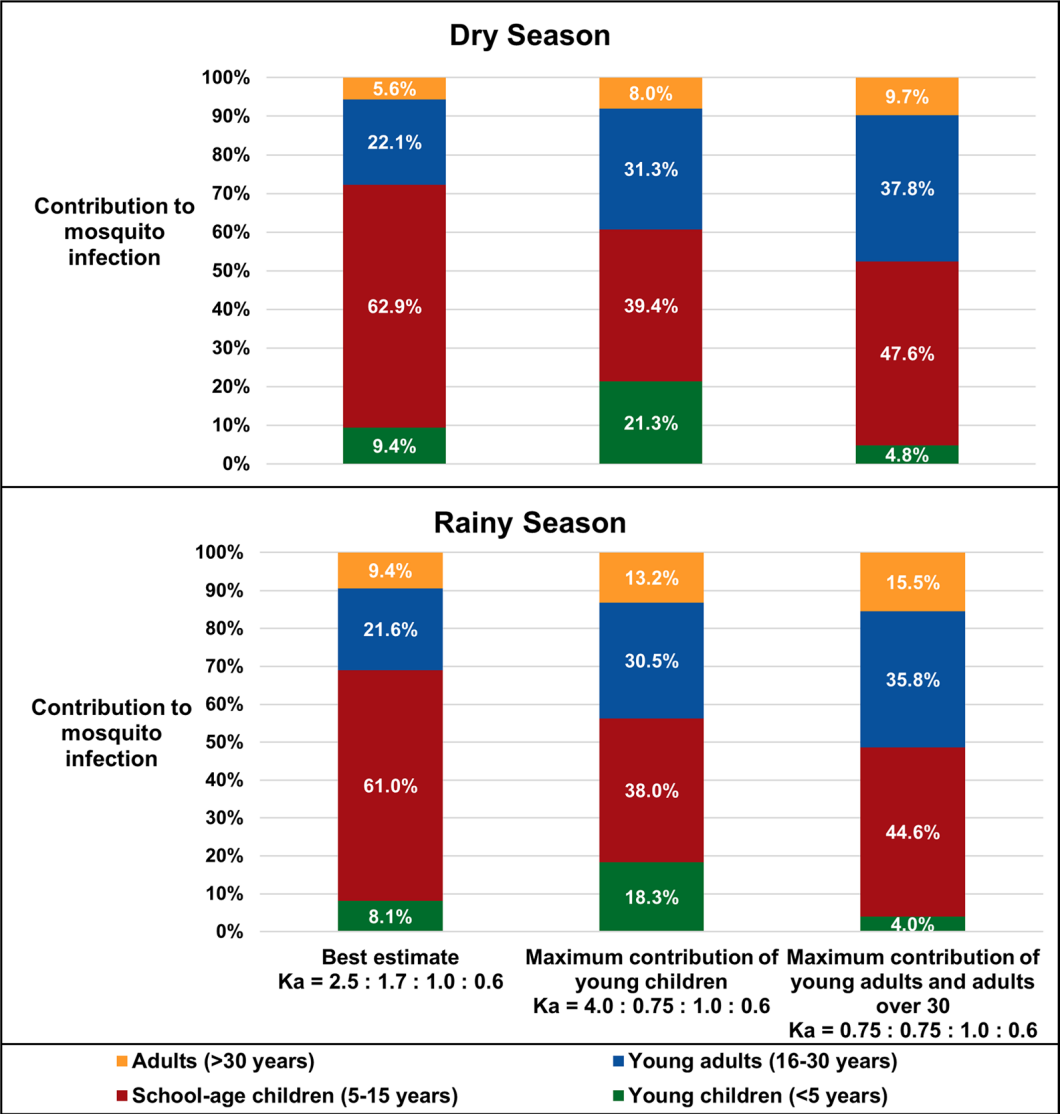
Extreme possibilities of reservoir contributions from sensitivity analysis of human-to-mosquito infectivity (*κ*_*a*_). Examples of extreme ends of the sensitivity analysis varying human-to-mosquito infectivity, as described in “Methods” and in Fig. 4

Given the uncertainty in the parameter value for infectivity by age, 4356 simulations were run per season with *κ*_<5_ and *κ*_*SAC*_ ranging from 0.75 to 4.0, incorporating mosquito biting heterogeneity and gametocyte density-dependent effects (Fig. 4). SAC were the largest contributors to transmission in southern Malawi under all age-dependent infectivity simulations, even for the most extreme conditions in which other age groups’ contributions were maximized (Fig. 5). SAC were responsible for > 50% of transmission if they were at least 20% more infectious than young adults during the dry season and 25% more infectious than young adults during the rainy season, regardless of the relative infectivity of U5s. Young adults (16–30 years) were the second largest contributors to new mosquito infections under all potential infectivity scenarios for both seasons, although in most scenarios they were responsible for a smaller proportion of new transmission than their 27.2% share of the population. Adults over 30, who comprise 24.7% of the population, were responsible for < 10% of transmission in all dry season simulations and most rainy season simulations (Figs. 4, 5).

On the other hand, the predicted transmission contribution of U5s was sensitive to both their infectivity and the infectivity of SAC. Children under five comprise 18.2% of the population, but could only have contributed ≥ 18.2% of new mosquito infections if they were highly infectious relative to all other age groups, particularly during the rainy season. During the rainy season, even in the most extreme simulation, U5s would only have contributed a maximum of 18.3% of transmissions (Fig. 5). In the majority of simulated conditions, they were responsible for < 10% of transmission. In all simulations, they contributed less to transmission than SAC and young adults.

## Discussion

Using recent molecular parasite data and reported ITN use in southern Malawi, this model estimated that SAC were responsible for > 60% of *P. falciparum* transmission to mosquitoes during both the dry and rainy seasons. The model estimated that young adults (16–30 years) were the second largest contributors to transmission; U5 children and adults over 30 years were each predicted to have caused < 10% of mosquito infections in most simulations. The proportion of mosquito infections attributable to each human age group did not differ considerably by season. Lacking human-to-mosquito infectivity data from skin or membrane feeding, sensitivity analyses were performed under a reasonable range of estimates of relative infectivity by age after accounting for gametocyte density distributions. If U5s are far more infectious to mosquitoes than all other age groups at a given gametocyte density, their estimated transmission contribution approached 20%, but SAC and young adults remained the largest and second largest contributors in these simulations. These findings may help explain *P. falciparum*’s persistence in the area despite considerable malaria control efforts in the past decade which targeted groups at high risk of disease—pregnant women and children under five.

Predictions about the transmission reservoir are sensitive to heterogeneity in mosquito biting, particularly in evaluating the contribution of U5s. Several prior lab studies of infectivity have made simple estimates of age-specific transmission contributions; however, those reports assumed homogeneous mosquito feeding [10–17]. The model presented here, like those of Stone et al. [21] and Gonçalves et al. [18], demonstrated that such a homogeneity assumption considerably overestimates the contribution of U5s and underestimates that of older age groups. In their mosquito blood meal source matching study, Gonçalves et al. [18] found that the degree of heterogeneity in mosquito biting by human age was different for their high and moderate transmission sites. The biting frequency ratio in the high transmission site was even more extreme than that used by Stone et al. and the model presented here, at 1:7:20 for U5s compared to SAC and adults 16 years and older, but the increase in biting frequency with age was moderate (1:2.3:2.1) and did not achieve statistical significance in the low/moderate intensity transmission site in Kenya. Given the sensitivity of transmission dynamics to this parameter, future research should better quantify heterogeneity in human exposure to feeding mosquitoes to more accurately identify key *P. falciparum* transmission reservoirs, and assess the factors which drive variation in this age-specific biting heterogeneity in different geographic settings.

The results presented here using recent empirical data from southern Malawi predict high transmission contribution from SAC, and are consistent with those of Stone et al. [21] which were based on generic sub-Saharan African estimates of parasite prevalence and population distribution. This suggests that the findings may be generalizable to other sub-Saharan African settings with high *P. falciparum* endemicity. A worksheet of the model calculations that other researchers can utilize in their specific contexts with, at a minimum, age-specific data on (1) population distribution; (2) estimated PCR prevalence; and (3) frequency of ITN use is provided as Additional file 3. Results should be interpreted with caution in settings with different malaria epidemiology, particularly those near elimination or where non-falciparum malaria is common.

A potential limitation of the study was the use of four compartments for gametocyte density, and the subsequent need to average the density-dependent transmission probability within each compartment. The estimation of parameters for each compartment based on Churcher et al. [24] resulted in a lower relative likelihood of transmission for the highest density categories compared to that applied in some other models [26]; however, the very small proportion of high density gametocyte carriers in this community-based population makes the model relatively insensitive to changes in the value selected for that compartment. This limitation would be critical, however, if the few high density gametocyte carriers are disproportionately likely to be fed upon by mosquitoes, essentially driving transmission as ‘superspreaders’. As seen in this model, transmission patterns may be highly sensitive to heterogeneity in blood feeding. More research matching blood meals to specific human sources is needed to understand mosquito feeding patterns in real-world settings. If it is discovered that a select few superspreaders are responsible for the majority of *Plasmodium* transmission, this would have significant limitations for targeting interventions aimed at control and/or elimination.

Further, the model is static and depends on measured prevalence data, without regard for the causes of differential prevalence of infection by human age. While this should not limit inferences about transmission contributions for the time-period studied, design of effective interventions will require better understanding of whether differences in infection prevalence are a result of differences in risk of infection, natural infection clearance time, frequency of anti-malarial treatment, or some combination of these.

## Conclusions

This model predicted that SAC and young adults were responsible for > 80% of all new mosquito infections in southern Malawi, with SAC contributing ~ 60% overall despite comprising only 30% of the population. A critical implication is that malaria elimination is unlikely in persistent settings like southern Malawi unless researchers and policy-makers develop interventions that can successfully reduce transmission from these groups, who are often asymptomatic and submicroscopically infected. More sensitive rapid detection methods and development of safe/effective drugs for transmission reduction are therefore fundamental to further progress toward malaria eradication.

## Additional files

**Additional file 1.** Additional detail on gametocyte testing methods. Text providing further detail on the molecular testing methods used for the detection of gametocytes.

**Additional file 2.** Summary of age-specific infectiousness data from membrane- and skin-feeding studies. Table providing abstracted data from a literature review of membrane- and skin-feeding studies, especially those that provided data on differential infectivity by age and any calculations of human contributions to the infectious or transmission reservoirs.

**Additional file 3.** Tool for site-specific model calculations. A spreadsheet that enables researchers and policy-makers to input data from their site into the model and estimate transmission contributions by age. Caution is recommended for the interpretation of this output in settings with malaria epidemiology that is very different than that of southern Malawi.

**Additional file 4.** Dry season model equations. Files for use with Berkeley Madonna modelling software that include model equations and dry season parameter estimates from this paper.

**Additional file 5.** Rainy season model equations. Files for use with Berkeley Madonna modelling software that include model equations and rainy season parameter estimates from this paper.

## Abbreviations

ICEMR: International Center of Excellence for Malaria Research
ITN: insecticide-treated net
LDH: lactate dehydrogenase
qPCR: quantitative polymerase chain reaction
qRT-PCR: quantitative reverse transcription polymerase chain reaction
SAC: school-age children
U5s: children under 5 years of age
