# Supplementary material for: Simulation models predict that school-age children are responsible for most human-to-mosquito Plasmodium falciparum transmission in southern Malawi
Source: Malar J. 2018 Apr 3;17:147. doi: 10.1186/s12936-018-2295-4 (PMC5883608; doi:10.1186/s12936-018-2295-4)
Supplement: Supplementary file 1 — Additional file 1. Additional detail on gametocyte testing methods. Text providing further detail on the molecular testing methods used for the detection of gametocytes. [file 12936_2018_2295_MOESM1_ESM.docx]

**Supplementary Material 1. Gametocyte testing methods**

All consenting participants over six months of age provided 50 μL whole blood samples from a finger prick preserved in 250 μL of RNAprotect® (Qiagen Inc., Valencia, CA) for quantitative reverse transcription-polymerase chain reaction (qRT-PCR). RNA samples were stored in a -80°C freezer within 24 – 48 hours of collection. Samples were thawed immediately before testing, when necessary.

qPCR targeting *P. falciparum* lactate dehydrogenase (LDH) DNA was run in duplicate to determine infection status. If either well was positive, qRT-PCR was performed on a sample of the person’s whole blood preserved in RNAprotect Cell Reagent (Qiagen) to detect expression of the mature gametocyte marker *Pfs25* [25, 30-32]. Preserved RNA samples were stored at −80 °C until thawed for RNA extraction and testing. After thawing, samples were spun in a micro-centrifuge at room temperature to obtain a pellet. RNA was extracted from the selected samples using RNeasy Plus Mini-Kits® (Qiagen Inc.,Valencia, CA) and treated with RNase-free DNase Sets®(Qiagen Inc., Valencia, CA) to eliminate parasite gDNA. The extracted RNA was stored at −80 °C until further analysis.

Reverse transcription and PCR were performed using TaqMan RNA-to-Ct 1-Step Kits (Applied Biosystems, Foster City, CA 94404. Part No. 4392938). The kit contains TaqManRT-PCR Mix (2X) and TaqMan RT Enzyme Mix (40X). Primer/probe mix was created for qRT-PCR by combining 18μL of 100uM forward primer stock (**5’GAA ATC CCG TTT CAT ACG CTT G 3’**) and 18μL of 100uM reverse primer stock (**5’AGT TTT AAC AGG ATT GCT TGT ATC TAA 3’**) for a final concentration of 900nM, 5μL of probe stock (100uM) (**6FAM TGT AAG AAT GTA ACT GTG GTA ACG GT TAMRA**) for a final concentration of 250nM, and 59μL of nuclease-free water. These mixes were stored in dark boxes at −20°C. Each well of the qRT-PCR reaction consisted of 10μL TaqMan RT-PCR Mix (2X), 0.5uL TaqMan RT Enzyme Mix (40X), 1μL of the primer/probe mix, 6.5μL of nuclease-free water, and 2μL of RNA sample. The assay was performed in duplicate.

The density of circulating gametocytes was automatically quantified by the RealTime PCR machine using a standard curve of gametocytes induced from Pf.2004 TdT *P. falciparum* (provided by the lab of Dr. Matthias Marti). The quantity of gametocytes in the standard curve was microscopically determined by three independent readers, then diluted using uninfected red blood cells to create a stock standard with a concentration of 50,000 gametocytes/μL. The standard was preserved in RNAprotect Cell Reagent and stored at -80ᵒC. The standard stock was diluted to 200 gametocytes/μL for the high density control and serially diluted (5-fold) to 5.12x10^-4^ for development of a standard curve that was run with each PCR plate and used to estimate the gametocyte density of the unknown samples. The PCR run was repeated if it did not achieve a sensitivity of at least 2.56 gametocytes/mL.
